# Selection of Aptamers for Use as Molecular Probes in AFM Detection of Proteins

**DOI:** 10.3390/biom13121776

**Published:** 2023-12-12

**Authors:** Maria O. Ershova, Amir Taldaev, Petr V. Konarev, Georgy S. Peters, Anastasia A. Valueva, Irina A. Ivanova, Sergey V. Kraevsky, Andrey F. Kozlov, Vadim S. Ziborov, Yuri D. Ivanov, Alexander I. Archakov, Tatyana O. Pleshakova

**Affiliations:** 1 Institute of Biomedical Chemistry, Pogodinskaya Str. 10/8, 119121 Moscow, Russia; motya00121997@mail.ru (M.O.E.); varuevavarueva@gmail.com (A.A.V.);; 2 A.V. Shubnikov Institute of Crystallography of Federal Scientific Research Centre “Crystallography and Photonics” of Russian Academy of Sciences, Leninsky Ave. 59, 119333 Moscow, Russia; 3 National Research Centre “Kurchatov Institute”, Akademika Kurchatova Square 1, 123182 Moscow, Russia

**Keywords:** AFM, aptamers, protein detection, CA125, bio specific interaction, SAXS

## Abstract

Currently, there is great interest in the development of highly sensitive bioanalytical systems for diagnosing diseases at an early stage, when pathological biomarkers are present in biological fluids at low concentrations and there are no clinical manifestations. A promising direction is the use of molecular detectors―highly sensitive devices that detect signals from single biomacromolecules. A typical detector in this class is the atomic force microscope (AFM). The high sensitivity of an AFM-based bioanalysis system is determined by the size of the sensing element of an atomic force microscope―the cantilever―the radius of the curvature of which is comparable to that of a biomolecule. Biospecific molecular probe–target interactions are used to ensure detection system specificity. Antibodies, aptamers, synthetic antibodies, and peptides can be used as molecular probes. This study has demonstrated the possibility of using aptamers as molecular probes for AFM-based detection of the ovarian cancer biomarker CA125. Antigen detection in a nanomolar solution was carried out using AFM chips with immobilized aptamers, commercially available or synthesized based on sequences from open sources. Both aptamer types can be used for antigen detection, but the availability of sequence information enables additional modeling of the aptamer structure with allowance for modifications necessary for immobilization of the aptamer on an AFM chip surface. Information on the structure and oligomeric composition of aptamers in the solution was acquired by combining small-angle X-ray scattering and molecular modeling. Modeling enabled pre-selection, before the experimental stage, of aptamers for use as surface-immobilized molecular probes.

## 1. Introduction

Biospecific complex formation between a probe and a target has been used in many biomedical devices, in particular diagnostic test systems, to detect specific pathological markers present in biological samples. Molecular probes can be immobilized on a surface, as in an enzyme-linked immunosorbent assay (ELISA), or occur in a solution.

Antibodies and, much less commonly, peptides and oligonucleotides (aptamers) have been used as molecular probes for protein targets. However, the use of antibodies is associated with a number of problems, in particular high cost of production and instability during storage [1].

Some of these problems can be solved by replacing natural antibodies with synthetic analogues―aptamers [2]. Aptamers are short single-stranded oligonucleotides that form complex molecular structures, which underlies their high affinity and specificity binding to targets [3]. Aptamers can be selected using an in vitro process called systematic evolution of ligands by exponential enrichment (SELEX), which was first developed by three independent groups in 1990 [4]. SELEX-selected aptamers can achieve high affinity and specificity binding to a variety of targets, including metal ions, small molecules, proteins, and even cells and tissues [5,6]. Aptamers have many advantages, including ease of synthesis, facile modification, and relative chemical stability. Therefore, aptamers are promising affinity reagents for biomarker detection [7].

**Figure 1:**
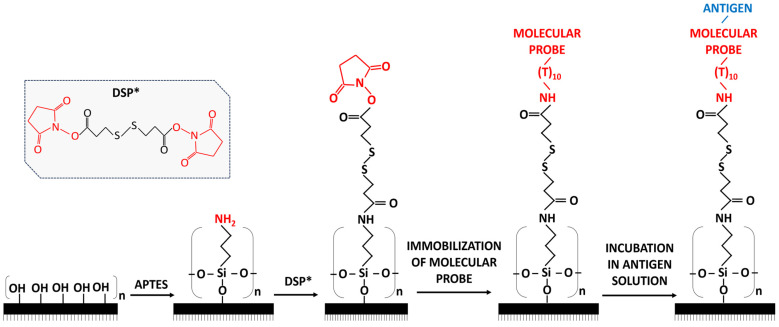
Schematic diagram of DSP cross-linker-based activation. (1) Mica surface before silanization; (2) APTES-modified mica surface ((aminopropyltriethoxysilane)); (3) mica surface after modification with DSP; (4) mica surface with an immobilized molecular probe; (5) mica surface after incubation in an antigen solution. *—chemical structure of DSP crosslinker.

Selection of aptamers for use as molecular probes is performed in two ways: the use of commercial aptamers and the selection of aptamer sequences from literature sources and their further synthesis. The most well-known companies producing aptamers are BasePair (Pearland, TX, USA), APTAMERLAB (Krasnoyarsk, Russia), and APTAGEN (Jacobus, PA, USA). An aptamer sequence is a company’s intellectual property, therefore, it is not always possible to make changes to the so-called linker that is required for covalent surface immobilization of the aptamer. Previously, we demonstrated the opportunity of using AFM chips with immobilized aptamers for biospecific fishing―selective binding of target protein molecules, which are present in the bulk of the test solution, to a small, specifically modified and sensitized surface. For example, this technique has been shown to be effective in detecting HCVcoreAg and Gp120 [8,9]. Surface sensitization refers to the immobilization of a probe for a target protein on the surface, which is a technique widely used in bioanalysis to ensure the specificity of protein detection. AFM analysis uses the scheme shown in Figure 1. To ensure covalent binding of aptamers, the surface of aminosilanized mica is modified with a dithiobis (succinimidyl propionate) (DSP) cross-linker specific to primary amino groups (Figure 1). This surface modification enables covalent immobilization of an aptamer that has a linker with a terminal amino group.

In previous studies, the incorporation of ten thymine bases and an amino group ((T_10_)–NH_2_) onto the 5′-end of a known sequence of an aptamer developed for therapeutic purposes allowed the aptamer to be used as a molecular probe for the detection of HCVcoreAg [8]. Studies on the detection of HCVcoreAg revealed that not all aptamers used as molecular probes were equally efficient towards the target antigen both in buffer solutions and in biological fluid samples. This study is the next step in our systematic research on the use of surface-immobilized aptamers for the detection of target proteins.

To date, a number of studies have reported affinity characteristics—dissociation constants, *K_d_*―for SELEX-selected aptamers. Presumably, the selection of aptamers for biospecific fishing systems should be based on *K_d_*: the lower this parameter, the better the aptamer’s affinity properties [8]. However, it should be noted that *K_d_* in most studies was determined for aptamers in a solution, which lacked modifications necessary for immobilization. It is necessary to determine how the required modification affects the affinity properties of aptamers. In this study, we performed experiments on protein detection using commercially available aptamers and aptamers synthesized based on sequences reported in open sources and containing the necessary modifications. The well-characterized CA125 protein, an important tumor marker associated with many human cancers and most widely used for the diagnosis and monitoring of ovarian cancer, was chosen as a model target protein [10,11]. This protein was also chosen because of its large molecular weight and, therefore, large protein globule size [12], which facilitated the interpretation of AFM data.

A study [13] reported the sequences of aptamers specific to the CA125 protein. *K_d_* was determined for SELEX-selected aptamers. The aptamers were immobilized onto a nitrocellulose membrane from solutions with different concentrations, and detection was performed on a DeNovix DS-11 spectrophotometer (Wilmington, DE, USA). The interactions between high affinity aptamers and appropriate protein domains were studied using molecular modeling.

For this study, we chose two sequences (apta#1 and apta#2) with the best *K_d_* [13] and the commercially available aptamer apta#3 (Table 1). The synthesis of apta#1 and apta#2 sequences [13] involved incorporation of the ((T_10_)–NH_2_) linker necessary for covalent immobilization of biomolecules onto aminosilanized mica. The commercial sample apta#3 involved incorporation of the ((T_10_)–NH_2_) linker that was added by BasePair Biotechnologies.

**Figure 2:**
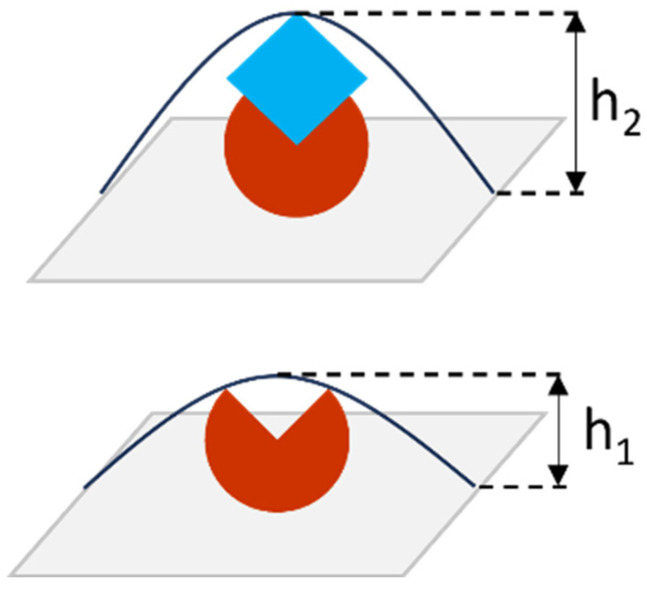
Schematic representation of protein complex formation on the surface: the red object is an immobilized molecular probe with height h_1_; the blue object is a target protein (antigen); a probe–protein complex with height h_2_ is formed on the surface during biospecific fishing. Antibodies, aptamers, and peptides can be used as the molecular probe. The height of objects is determined by AFM measurements―scanning of the surface and analysis of its topography.

The efficiency of selected aptamers as molecular probes was evaluated in a series of experiments on CA125 detection by biospecific fishing on a surface with immobilized aptamers. In the case of AFM analysis, the detected parameter is the height of objects visualized on the surface. Measuring the height of objects (protein molecules) before and after biospecific interaction is used to evaluate the formation of protein complexes (Figure 2). When the probe–target complex is formed, height h_2_ is greater than h_1_, where h_1_ is the object’s height after immobilization of the molecular probe onto the surface, and h_2_ is the object’s height after incubation of the AFM chip in the antigen solution. The height of visualized objects is determined by processing AFM data obtained from scanning of the surface covered with a large number of objects (at least hundreds), which is performed in different areas of the AFM chip.

Experimental design: At the first stage, the sizes of CA125 biomolecules and aptamers sorbed on freshly cleaved mica were assessed using AFM data. At the second stage, the effect of apta#1 and apta#2 modifications was assessed using molecular modeling and small-angle X-ray scattering (SAXS). At the third stage, the efficiency of aptamer–protein complex formation due to biospecific interactions was evaluated.

## 2. Materials and Methods

### 2.1. Target Protein

We used the recombinant CA125 protein (R&D Systems, Minneapolis, MN, USA) with a molecular weight of 110 kDa. Protein purity was more than 90% according to SDS-PAGE. The lyophilized protein was reconstituted in 250 μL of a buffer solution (PBS), pH 7.4. The initial concentration of the protein solution was 2.2 μM. PBS was obtained from JSC Vector Best (Novosibirsk, Russia).

### 2.2. Aptamers

In this study, we used solutions of anti-CA125 aptamers with known sequences (apta#2.26 [13]—apta#1; apta#2.43 [13]—apta#2). The sequences of apta#1 and apta#2 are given in Table 1. The aptamers were modified with ten thymine bases and an amino group, (T_10_)-NH_2_, at the 5′-end to allow covalent immobilization on the AFM chip surface. The aptamers were synthesized at Evrogen (Moscow, Russia).

An anti-CA125 aptamer, apta#3 (ATW0055, BasePair Biotechnologies, Pearland, TX, USA), was used in the study. The sequence of this aptamer is the intellectual property of BasePair Biotechnologies. The aptamer was modified with ten thymine bases and an amino group, (T_10_)-NH_2_, at the 5′-end. The aptamer’s length is 70 bases. The aptamer’s solution concentration is 50 μM. According to the manufacturer, the dissociation constant is 33.8 ± 7.9 nM.

### 2.3. Buffer Solutions

AFM samples were prepared using Dulbecco’s modified phosphate buffered saline (PBSD) 10 mM, pH 7.4 (Pierce, Washington, DC, USA).

To prepare buffer solutions and wash samples, we used deionized water produced using a Millipore Simplicity UV deionizer (Millipore, Molsheim, France).

### 2.4. Other Reagents

DSP (Pierce, Waltham, MA, USA) was used to activate aminosilanized mica; dimethyl sulfoxide (DMSO) (99.9%, Sigma, St. Louis, MO, USA) was used to prepare a DSP cross-linker solution. Ethyl alcohol (Reakhim, Moscow, Russia) was used to prepare a DSP cross-linker solution and wash chips after surface activation. Emulgen 913 (Kao Atlas, Japan), a 0.01% solution in deionized water, was used to wash the surface of chips after the biospecific fishing procedure. Mica (SPI, Washington, DC, USA) modified with 3-aminopropyltriethoxysilane (APTES) was used as an AFM chip substrate.

All used solutions (aqueous emulgen solution, ethyl alcohol, buffer solutions) were filtered using Amicon Ultra centrifugal filters 3 kDa MWCO (Millipore, USA). The content of particles in the filtered solutions was monitored by AFM.

Laboratory equipment and consumables: thermal shaker (Thermomixer comfort, Eppendorf, Hamburg, Germany), centrifuge and vortex CM-70M-09 (ELMI, Rīga, Latvia). Disposable plastic utensils: 0.2, 1.5, and 50 mL microtubes; Petri dishes; tips for automatic pipettes.

### 2.5. Atomic Force Microscopy

AFM images were acquired using Aura Integra and Titanium atomic force microscopes (NT-MDT, Moscow, Russia). The data were obtained in tapping mode in air, which is a scanning mode where the cantilever acts as delicately as possible on biomacromolecules visualized on the surface. AFM NSG10 cantilevers (“TipsNano”, Zelenograd, Russia) with a reflective gold surface were used. The cantilever stiffness constant ranged from 3.1 to 37.6 N/m, and the typical resonant frequency ranged from 140 to 390 kHz. The cantilever radius of curvature was no more than 10 nm.

AFM images were captured using software NOVA PX 3.5.0 rev. 20364 (NT-MDT, Russia). The AFM was adjusted by changing basic parameters. AFM images were processed using Image Analysis 3.5.0.20345 Trunk software (NT-MDT, Russia).

Further processing of scanning data was performed using special software (Recognite10) developed at the Institute of Biomedical Chemistry. Statistical data processing was carried out using Microsoft Excel and involved calculating the object height distribution function, *ρ(h)*, and counting the number of objects per 400 μm^2^ of area. For experiments performed twice or more, the reported values include standard deviations calculated in Microsoft Excel. To calculate the absolute object number, the number of objects per 400 µm^2^ of area (16 AFM image of 25 µm^2^ each) was calculated using the formula:

(1) $$N_{400}=\frac{N*400}{n*S_{fr}}$$

where *N*_400_ is the normalized number of objects per scanning area of 400 μm^2^; *N* is the number of objects detected in one experiment; *n* is the number of AFM images in one experiment; and *S_fr_* is the AFM image area in µm^2^.

Additionally, the object height distribution density, *ρ(h)*, was used to analyze object heights:

(2) $$\rho \left(h\right)=\frac{N_{h}}{N}*100\%$$

where *N_h_* is the number of objects with height *h* detected in one experiment and *N* is the total number of objects detected in one experiment.

### 2.6. Small-Angle X-ray Scattering (SAXS)

High-concentration solutions were used in SAXS experiments. Experimental SAXS data were acquired using the BioMUR beamline (Kurchatov Institute, Moscow, Russia) [14]. The sample-to-detector distance was 700 mm, covering the range of the momentum transfer 0.2 *< s <* 6.0 nm^−1^ (*s* = 4π sin*θ*/*λ*, where 2*θ* is the scattering angle and *λ* is the X-ray wavelength equal to 0.1445 nm). The exposure time was 10 min. Measurements were carried out at a temperature of 25 °C. Sample and buffer solutions were placed into thin quartz capillaries of 2 mm in diameter. Silver behenate data were used to calibrate the angular axis. Next, the data for apta#1 and apta#2 samples (3, 5, 10, and 15 mg/mL solutions) were acquired.

Signals detected by a two-dimensional pixel detector Pilatus 3M 1M provided information on X-ray scattering by biomolecules. Next, the data were processed using the ATSAS 3.0.4 software [15] package. A one-dimensional scattering curve was created using FIT2D (module of ATSAS 3.0.4 software) [16] that averaged two-dimensional scattering patterns over the radial direction, which was possible due to the isotropic nature of the scattering. PRIMUS (module of ATSAS 3.0.4 software) [17] was used for background (deionized water) subtraction from each sample. The pair distribution function for distances between intramolecular scattering centers was constructed using GNOM (module of ATSAS 3.0.4 software) [18]. A spatial ab initio model was generated using DAMMIF (module of ATSAS 3.0.4 software) [19]. To represent a spatial distribution of the electron density of a molecule, the search volume of the particle was filled by a set of densely packed small-sized beads. These beads were used to build a sphere with a diameter equal to the maximum molecule size. Next, a bead model of the molecule was generated, which was calculated by minimizing the discrepancy *χ*^2^ between the scattering intensity calculated from the current model and experimental data. The latter was calculated using the formula:

(3) $$\chi^{2}=\frac{1}{N-1}\sum_{j=1}^{N}{\left[\frac{I_{exp}(s_{j})-cI_{calc}(s_{j})}{\sigma (s_{j})}\right]}^{2}$$

where *N* is the number of experimental data points and *c* is the scaling factor overlaying the compared scattering curves according to the least squares criterion and calculated by the formula:

(4) $$c=\left[\sum_{j=1}^{N}\frac{I_{exp}(s_{j})I_{calc}(s_{j})}{\sigma (s_{j})}\right]{\left[\sum_{j=1}^{N}\frac{{I_{exp}(s_{j})}^{2}}{\sigma (s_{j})}\right]}^{-1}$$

where *I_exp_(s_j_)* and *I_calc_(s_j_)* are the experimental and calculated scattering intensity, respectively, and *σ(s_j_)* is the experimental error corresponding to the momentum transfer *s_j_.*

The resulting ab initio bead model was generated by averaging 20 independent DAMMIF models using DAMAVER (module of ATSAS 3.0.4 software) [20].

The obtained data were then compared with the molecular modeling data.

### 2.7. Molecular Modeling

Molecular modeling was performed in three steps. At the first step, the secondary structure of the aptamers was predicted. The second step involved calculating the spatial structure of the aptamers. At the third step, the most populated aptamer conformations were identified using molecular dynamics.

The secondary structures of the aptamers were predicted using the Mfold service [21]. The nucleotide sequence, temperature (25 °C), and sodium and magnesium ion concentrations were specified as input data [22]. Next, the structure with the lowest Gibbs [23] free energy was selected for each aptamer.

The spatial structures of the aptamers were predicted using the RNA Composer web service [24,25]; however, the service predicts RNA structures only. The input data included the nucleotide sequence and the secondary structure in the dot-bracket format produced in Mfold. After prediction, RNA was converted back to DNA in BIOVIA Discovery Studio Visualizer 4.5 by replacing ribose with deoxyribose and uracil with thymine.

Molecular dynamics (MD) were simulated using GROMACS 2020.4 software [26]. The AMBER14SB force field [27] with CUFIX OL15 corrections for DNA was selected. This selection was based on literature data on the acceleration of protein and nucleic acid folding in the AMBER14SB field compared with that in the standard AMBER force field series. MD simulations were run in a solvent with periodic boundary conditions (TIP3P water model). Each initial structure was placed at the center of a cubic cell of sufficient size to ensure that the minimum distance to period boundaries was 1.0 nm. Some water molecules were replaced by Mg^2+^ and Cl^–^ ions, with the overall net charge being neutral. Next, the energy of the system was minimized using the conjugate gradient method (5000 iterations) and annealed for 5 ns with an increment of 1 fs. In this case, a Berendsen barostat [28] and a V-rescale thermostat [29] were used. A Parrinello–Rahman barostat [30] and a V-rescale thermostat [29] were used for productive MD. Simulations were carried out at a temperature of 298 K and a pressure of 1 bar. The cut-off for non-covalent interactions was set to 1.4 nm. Trajectories were processed using the trjconv, trjcat, rms, cluster, and SAXS modules built into the GROMACS package. The clustering procedure was performed using the gromos method [31]; the clustering cut-off value was selected empirically. The MD simulation details are shown in Table 2.

The most populated aptamer conformations calculated during molecular modeling were compared with experimental SAXS data produced by the ATSAS 3.0.4 software package using CRYSOL module [32] and visualized using Open-Source PyMOL 2.5.0. The most probable model was selected using the *χ*^2^ criterion, which evaluates the discrepancy between the theoretical intensity calculated from a molecular model and experimental SAXS data.

## 3. Results and Discussion

### 3.1. AFM Visualization of the Protein and Aptamers Sorbed on Freshly Cleaved Mica

Atomic force microscopy enables exploration of single biomolecules. For efficient AFM analysis, biomolecules should be fixed to the surface. Fixation is implemented through both sorption (non-covalent immobilization) and covalent bond formation between active surface groups and biomolecule sites [33]. Several substrates are currently used in AFM: freshly cleaved mica and highly oriented pyrolytic graphite (HOPG).

At the first stage of experiments, biomolecules were sorbed onto the surface of freshly cleaved mica. This stage is necessary to assess the aggregated state of biomolecules and the contribution of nonspecific molecules that may occur in solution after synthesis and purification. In this experimental series, the CA125 protein and commercial and synthesized aptamers were sorbed onto the surface.

**Figure 3:**
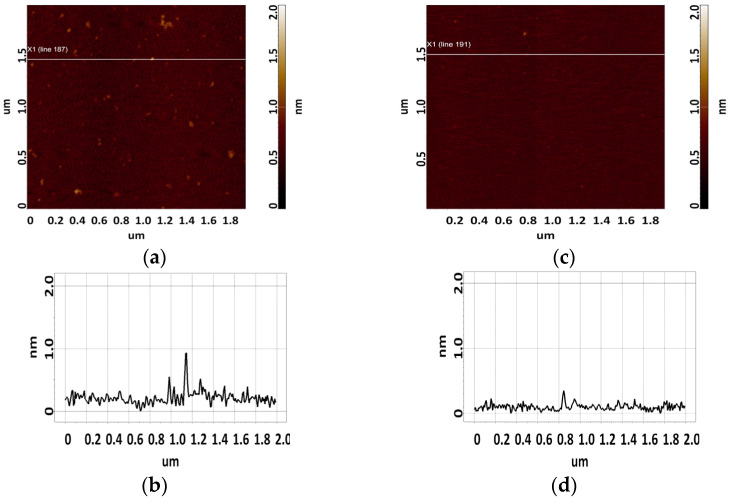
Typical AFM images of the surface of freshly cleaved mica after sorption of the CA125 protein from a 2.2 μM solution (**a**), and the surface of freshly cleaved mica after incubation in a PBS buffer solution used to dissolve the CA125 protein (control, c). Sections (**a**,**c**) corresponding to the white line in (**b**,**d**), respectively.

The CA125 protein was sorbed from a 2.2 μM solution. Single objects of 0.8 to 1.4 nm in height were visualized on the surface using AFM. The sorbed visualized objects were attributed to CA125 molecules because no such objects were detected in control experiments (visualization of the surface after incubation in protein-free buffer) (Figure 3b). A typical AFM image of the surface after sorption of the CA125 antigen is shown in Figure 3a.

Magnesium ions (or other bivalent metal ions) are necessary for efficient sorption of aptamers onto the surface of freshly cleaved mica because the charge of aptamer molecules is strongly negative, and the charge of freshly cleaved mica is negative too. Due to their charge, magnesium ions improve the sorption of aptamers onto the surface. Additionally, magnesium ions are key for the folding of aptamers. Sorption of aptamers onto freshly cleaved mica and subsequent AFM imaging enable evaluating the sizes of single aptamer molecules. At this stage, the use of a freshly cleaved mica instead of a silanized one, which is used for covalent immobilization, minimizes the contribution of substrate to the height of the molecule.

Determination of the height of aptamers sorbed on the surface is necessary for comparison with molecular modeling data and to assess the efficiency of monolayer formation after immobilization of aptamers on a silanized mica surface. At this stage, experiments involved only apta#1 and apta#2 samples because they were synthesized using published sequences, which enabled working with both concentrated and dilute solutions.

**Figure 4:**
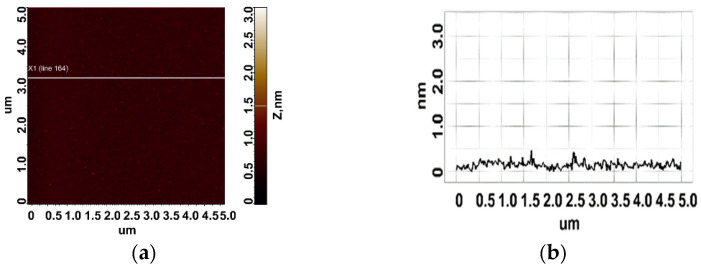
Control experiment: a typical AFM image of a freshly cleaved mica surface after incubation in an MgCl_2_ solution (**a**). Section (**b**) corresponds to the line in (**a**).

To determine the height of aptamers, they were sorbed onto freshly cleaved mica from 10 μM, 1 μM, and 0.1 μM aptamer solutions. Different solution concentrations were used to increase the reliability of data and evaluate the contribution of an aggregated state of molecules to the results of AFM imaging. The control in this series of experiments included AFM imaging of the surface after incubation of freshly cleaved mica in a MgCl_2_ solution. The visualization results are shown in Figure 4. As can be seen in Figure 4, no objects with a height of more than 0.5 nm were detected on the surface.

**Figure 5:**
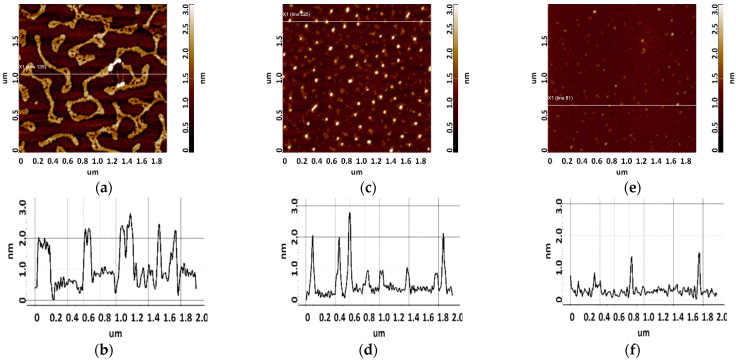
Visualization of apta#1 on freshly cleaved mica. Typical AFM images after sorption of biomolecules from 10 μM (**a**), 1 μM (**c**), and 0.1 μM (**e**) solutions containing magnesium. Sections (**b**,**d**,**f**) correspond to lines in (**a**,**c**,**e**).

**Figure 6:**
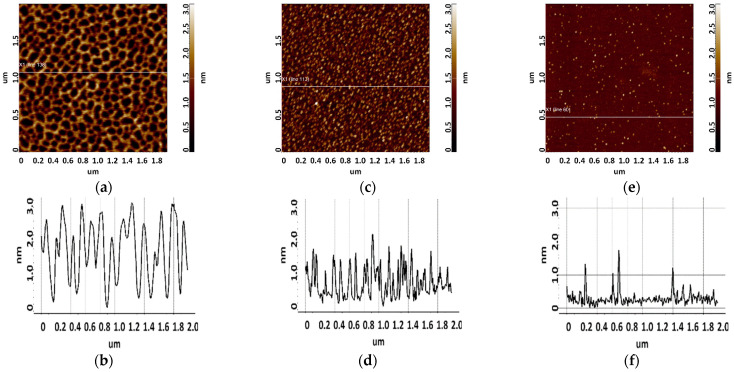
Visualization of apta#2 on freshly cleaved mica. Typical AFM images after sorption of biomolecules from 10 μM (**a**), 1 μM (**c**), and 0.1 μM (**e**) solutions containing magnesium. Sections (**b**,**d**,**f**) correspond to lines in (**a**,**c**,**e**).

The results of surface visualization after incubation in solutions containing aptamers are presented in Figure 5 and Figure 6.

As seen from Figure 5, layered elongated objects of no more than 3 nm in height were visualized on the mica surface after sorption of apta#1 from a 10 μM solution. Upon sorption of apta#2 (Figure 6) from a solution of the same concentration, a layer of the same height was observed on the surface. Layered structures indicate an aggregated state of biomolecules in a 10 μM solution.

Upon sorption of aptamers from a 1 µM solution, single objects were visualized on the surface; the height of objects in the case of apta#1 and apta#2 was no more than 3 nm and 2.2 nm, respectively. At this solution concentration, biomolecules are predominantly sorbed as individual objects rather than layers, but their height indicates an aggregated state because this height corresponds to biomolecules with a much higher molecular weight than that of the aptamer, as in the case of CA125 protein visualization (Figure 3). It is worth noting that the height and lateral dimensions of objects were larger in the case of apta#1 than in the case of apta#2.

**Figure 7:**
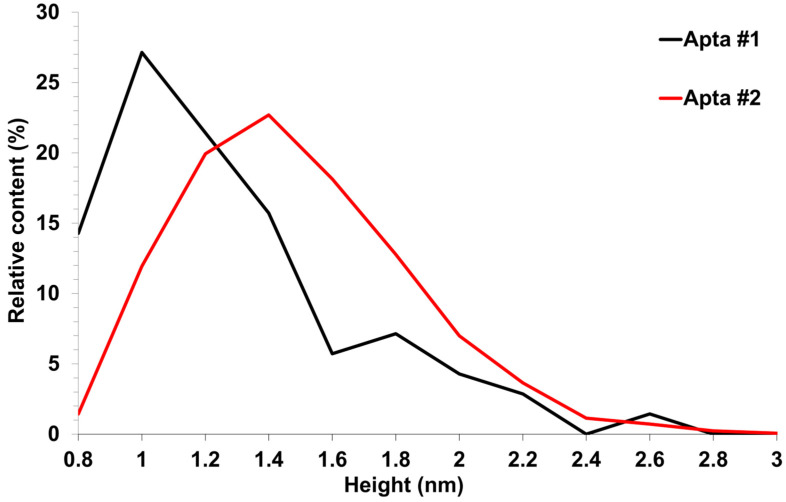
AFM visualization of aptamers sorbed on freshly cleaved mica from 0.1 μM solutions. Relative distribution of visualized objects by height: apta#1 (black line) and apta#2 (red line).

Upon sorption of aptamers from a 0.1 μM solution, a fewer objects were visualized on the surface. The AFM imaging results were statistically processed, and Figure 7 shows the relative distribution of visualized objects by height. As seen from Figure 7, the height of visualized objects was 1.0 ± 0.2 nm in the case of apta#1, but there were also objects with a height of 1.8 ± 0.2 nm. In the case of apta#2, the height of visualized objects was 1.4 ± 0.6 nm. Objects of more than 1 nm in height indicated the presence of aggregated structures, along with the monomeric form, in aptamer solutions. Thus, in the case of apta#2, AFM data indicated the presence of molecular aggregates in the solution even at the lowest concentration of 1 μM that were sorbed on the surface. These results suggested the aggregation of aptamer molecules in highly concentrated solutions. All objects visualized on the surface were attributed to aptamer molecules because no objects were detected in control experiments (visualization of the surface after incubation in a MgCl_2_ solution free of aptamer molecules) (Figure 4).

In the case of apta#3, no objects were detected on freshly cleaved mica even for a 50 μM solution, which prevented the estimation of the sizes of biomolecules. The visualization results corresponded to those of control experiments (Figure 4). Because the composition of the commercial aptamer solution was not known in detail, we suggest that some components may have prevented sorption of biomolecules onto the surface even in the presence of magnesium ions.

### 3.2. Comparative Analysis of Molecular Modeling and SAXS Data

Data on the oligomeric state of aptamers were obtained during the second stage using a combination of molecular modeling and SAXS. The structural features of aptamers apta#1 and apta#2 were analyzed. Apta#3 was not used because of the lack of information about its sequence, which prevented molecular modeling.

The secondary and tertiary structures of aptamers in an aqueous environment were determined using molecular modeling. The absence of ions was due to the fact that aptamers were immobilized on the surface of activated aminosilanized mica from an aqueous solution. The secondary structure was predicted using the MFold server (at 25 °C). Conversion to the spatial structure was performed using the RNAComposer server. Because this server predicts the structure of RNAs only, ribose and uracil in the sequence under study were replaced by deoxyribose and thymine, respectively, using Discovery Studio Visualizer 4.5 software.

The system was minimized in energy and then annealed. The obtained data were used to perform productive molecular dynamics that provided 43 and 18 putative conformations in the case of apta#1 and apta#2, respectively. The most probable conformation for each aptamer was selected by comparing theoretical scattering curves calculated from the resulting models with experimental SAXS data.

To compare SAXS data with apta#1 and apta#2 structures generated by molecular modeling, we used CRYSOL module of ATSAS 3.0.4 software [32] that calculated the radius of gyration (*R_g_*) and maximum particle size (*D_max_*) for each possible conformation, the theoretical scattering curve from the model, and the discrepancy value (*χ*^2^) evaluating the difference between a molecular model and the structure of apta#1 or apta#2 investigated in SAXS experiments. SAXS data from 15 mg/mL (1.2 mM) apta#1 and 10 mg/mL (0.73 mM) apta#2 solutions were analyzed because these concentrations were characterized by the highest signal-to-noise ratio and no concentration dependence was observed.

Based on experimental SAXS data for apta#1, *R_g_* (3.3 ± 0.1) nm and *D_max_* (11.5 ± 0.5) nm were estimated. The data for all apta#1 models are summarized in Table 3.

The closest model out of the generated set was model 11 that was characterized by a discrepancy value of *χ*^2^ = 4.9. This value indicates that the model did not fully fit the experimental data but was the most probable one among those generated using molecular dynamics. There were probably several apta#1 conformations in the solution that were characterized by equal interconformational transition probabilities, but they all most likely had similar structural parameters, such as *R_g_* and *D_max_.* Table 3 demonstrates that as the radius of gyration and the particle size increased, the discrepancy value *χ*^2^ became greater, i.e., the difference between the theoretical intensity calculated from a model and experimental SAXS data increased. Smaller *R_g_* and *D_max_* values, which meant a more compact particle size, also led to an increase in *χ*^2^ values.

**Figure 8:**
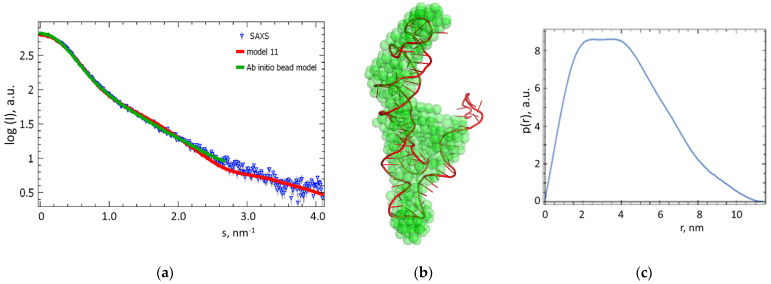
Comparison of experimental SAXS data with an apta#1 aptamer model from molecular dynamics. (**a**) Experimental SAXS data for apta#1 (blue triangles) and scattering curves calculated from an ab initio bead model (green line) and apta#1 model 11 (red line) generated using molecular dynamics). (**b**) Three-dimensional ab initio visualization of ab initio bead model obtained from SAXS data for an apta#1 solution (green spheres) and apta#1 model 11 generated using molecular dynamics (red chains). Models were overlapped using Supcomb of ATSAS 3.0.4 [34]. (**c**) Distance distribution function p(r) calculated from experimental SAXS data using GNOM module of ATSAS 3.0.4 software [18].

For the apta#1 model with the lowest *χ*^2^ value, plots with the best fits to the experimental SAXS data (Figure 8a) and three-dimensional visualization by overlaying (Supcomb from ATSAS 3.0.4 [34]) the molecular model with the ab initio three-dimensional shape of apta#1 reconstructed from SAXS data (Figure 8b) demonstrating good agreement between the models are presented.

Based on SAXS data for apta#2, *R_g_* (5.9 ± 0.1 nm) and *D_max_* (19.3 ± 0.5 nm) were estimated. During productive molecular dynamics, 18 possible apta#2 conformations were generated that were compared with experimental SAXS data. The mean maximum diameter *D_max_* calculated over 18 possible conformations was 10.0 ± 0.5 nm. In an aqueous solution, apta#2 presumably formed high-order oligomers that were significantly larger than monomer molecules.

### 3.3. Evaluation of the Efficiency of Aptamer–Protein Complex Formation on an AFM Chip Surface

The third stage involved biospecific fishing and AFM assessment of the efficiency of complex formation between an aptamer immobilized on the surface and the target protein CA125 in a solution. The commercial (apta#3) and synthesized (apta#1 and apta#2) aptamers (Table 1) immobilized on activated aminosilanized mica (AFM chip) were used in the study. AFM chips were incubated in a CA125 protein solution. The solution concentration was 1 nM in the case of apta#3 and 1 nM, 0.1 nM, or 0.01 nM in the case of apta#1 and apta#2. AFM analysis was performed before and after incubation with the protein.

**Figure 9:**
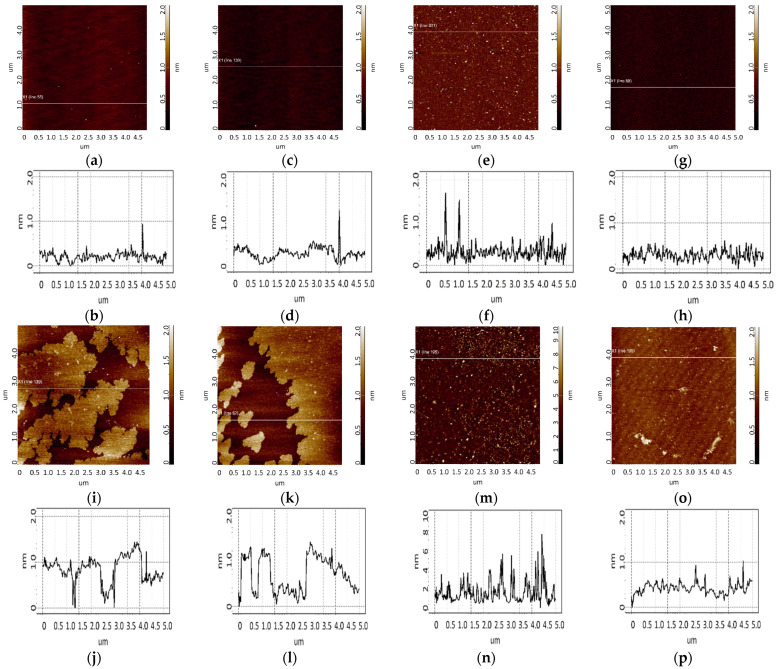
Detection of CA125 (1 nM solution) using chips with immobilized aptamers. Typical AFM images after immobilization of aptamers (**a**,**c**,**e**,**g**) and after incubation in a test antigen solution (**i**,**k**,**m**,**o**). The chip comprised immobilized apta#1 (**a**,**i**), apta#2 (**c**,**k**), apta#3 (**e**,**m**), and an aptamer-free control area outside the sensing zone (**g**,**o**). Sections (**b**,**d**,**f**,**h**,**j**,**l**,**n**,**p**) correspond to the lines in images (**a**,**c**,**e**,**g**,**i**,**k**,**m**,**o**).

**Figure 10:**
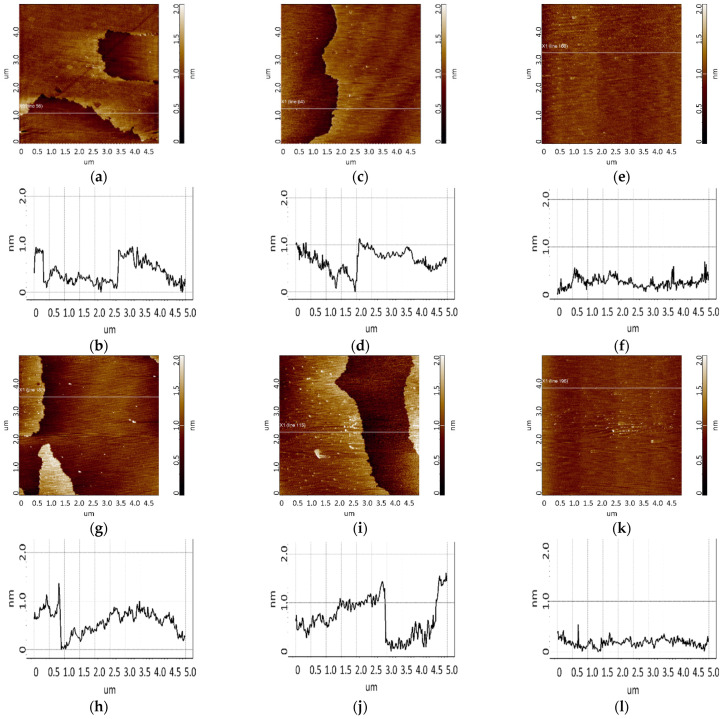
Detection of CA125 in 0.1 nM (**a**,**c**,**e**) and 0.01 nM (**g**,**i**,**k**) solutions using chips with immobilized aptamers. Typical AFM images after incubation in a test antigen solution (**a**,**c**,**e**,**g**,**i**,**k**) and sections (**b**,**d**,**f**,**h**,**j**,**l**) corresponding to the lines in images. The chip comprised immobilized apta#1 (**a**,**g**), apta#2 (**c**,**i**), and an aptamer-free control area (**e**,**k**).

The surface with immobilized aptamers before and after incubation in the CA125 solution is shown in Figure 9 and Figure 10.

**Figure 11:**
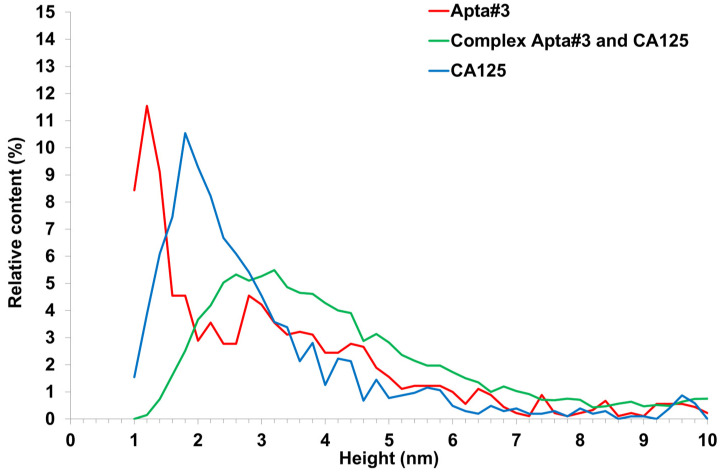
Relative distribution (A) of visualized objects on an AFM chip surface after immobilization of apta#3 (red line); after apta#3–antigen complex formation (green line); after sorption of the CA125 antigen onto the surface of freshly cleaved mica (blue line).

Efficient detection requires immobilization of aptamers in the form of a monolayer, which increases the likelihood of complex formation upon collision of the target protein with the surface during incubation. A study [35] has reported the sorption of one aptamer per 12.9 nm^2^ of a silanized surface. This is related to the fact that one APTES molecule interacts with three OH groups exposed on the silicon surface. Thus, one amino group is localized over an area of 12.9 nm^2^. Aptamers comprise an amino linker group that is involved in covalent binding to the aminosilanized surface through a DSP cross-linker. As seen from Figure 9a,c,e, a small number of objects of up to 1.5 nm in height were visualized on an aptamer-immobilized surface, with surface roughness being 0.2–0.3 nm. In this case, in the control area (Figure 9g), there were no compact objects, and the roughness was 0.1 nm. The objects and the chip surface roughness in the area with immobilized aptamers enabled assessing the efficiency of immobilization: aptamer molecules occurred on the surface presumably as a layer because compact objects were quite rare (no more than 10 per 25 µm^2^ scan). The largest number of compact objects was observed in the case of apta#3 immobilization. AFM scanning data for this sample were plotted as a height distribution function of visualized objects *ρ*(h) (Figure 11, red curve). As seen from the figure, after immobilization of apta#3, objects were present on the surface, most of which corresponded to the distribution maximum of 1.2 ± 0.2 nm.

Visualization of the surface after CA125 biospecific fishing is presented in Figure 9i,k,m and Figure 10a,c,e,g,i,k. As seen from the figures, in all cases of chips with immobilized apta#1 and apta#2, there were layered objects with a height of 1 to 1.5 nm that were present on the surface after incubation in the antigen solution with concentrations of 1 nM (Figure 9i,k), 0.1 nM (Figure 10a,c), or 0.01 nM (Figure 10g,i). In the case of apta#3, only the solution with the highest antigen concentration of 1 nM was analyzed; after incubation with the solution, compact objects of up to 6 to 7 nm in height were visualized on the surface. In all control experiments, neither layered nor compact objects were detected on the aptamer-free surface (Figure 9o and Figure 10e,k). Therefore, all objects visualized on the aptamer-immobilized surface after incubation in test CA125 solutions of various concentrations may be attributed to target protein molecules. In all cases, the protein was successfully detected using chips with immobilized aptamers because aptamer–protein complexes were visualized on the surface.

A plot of the height distribution function of visualized objects was created for apta#3 only, when compact objects were visualized on the surface. Apta#1 and apta#2 data could not be processed in this way because layered objects were present on the surface. The result of data processing is presented in Figure 11 (green curve). This figure also shows data obtained at the first stage upon visualization of the CA125 protein sorbed on the surface of freshly cleaved mica (Figure 11, blue curve). As seen from Figure 9e,f after incubation of the AFM chip with immobilized apta#3 in an antigen solution, objects of more than 1.4 nm in height (88% of the total objects) were predominantly visualized. The shift of the *ρ* (h) plot to the right upon visualization of the surface after incubation in a test solution (compare the green curve versus the red and blue) also indicated complex formation on the surface because the height of these complexes should have been higher than that of the immobilized probe molecules (Figure 2).

AFM visualization revealed that all aptamers tested in the study could be used to detect CA125 in a solution. However, the protein was detected only in a nanomolar solution in the case of commercial apta#3 and in solutions of lower concentrations (0.1 and 0.01 nM) in the cases of apta#1 and apta#2. It should be noted that *K_d_* for apta#3 was lower than that for apta#1 and apta#2 (Table 1). Probably, the affinity properties of apta#3 were higher during the selection of aptamers, but its affinity decreased due to immobilization onto the surface or introduction of an additional linker into the sequence. The structural features of the apta#3 aptamer could not be identified because its sequence was unknown. In the case of apta#1 and apta#2, modeling using SAXS data revealed the structural features of molecules and their aggregates in the solution. Estimation of apta#1 and apta#2 sizes using molecular modeling and their comparison with SAXS data revealed that the size of the apta#1 molecule in an aqueous solution at a temperature of 25 °C was 10.4 nm along the longest side. Differences in aptamer sizes in an aqueous solution, measured by SAXS and AFM, may be due to the impact of the substrate upon immobilization of biomolecules on the surface. According to SAXS data, apta#2 in an aqueous solution occurred in a highly aggregated state, which was confirmed by AFM data upon visualization of the aptamer sorbed onto freshly cleaved mica. High aggregation may affect the affinity properties of a probe in a solution. In our case, immobilization presumably precluded aggregation of the aptamer on the surface, and the aptamer binding centers remained accessible for complex formation.

## 4. Conclusions

In bioanalysis systems aimed at protein detection, the type of used molecular probes plays an important role. This study has demonstrated the possibility of using aptamers to detect the CA125 tumor marker. The protein was detected in a nanomolar solution using AFM chips with immobilized aptamers. The study has demonstrated the possibility of using both a commercial aptamer and aptamers synthesized based on known sequences and modified for covalent immobilization. Both aptamer types may be used for antigen detection, but the availability of sequence information enables additional modeling of the aptamer structure with allowance for modifications necessary for immobilization of aptamers on an AFM chip surface. Modeling enables pre-selection, before experiments, of aptamers for use as molecular probes immobilized on the surface. The study has demonstrated that one of the test aptamers is prone to aggregation, which may affect the affinity properties due to blockage of binding sites for a target protein in an aggregated structure. The combination of techniques exploited in this study may be successfully used to select optimal probes for other proteins, in particular disease biomarkers.

The results of this study may be used in other bioanalysis systems based on biospecific probe–target recognition, such as ELISA.

## Figures and Tables

**Table 1:** CA125-specific aptamers.

Name	Sequence	*K_d_*, nM
apta#1	TAG GGA AGA GAA GGA CAT ATG ATT TTA GGG AAG AGA AGG ACT TTT ATG CCG CCT TGA CTA GTA CAT GAC CAC TTG A [13]	166
apta#2	TAG GGA AGA GAA GGA CAT ATG ATG ATC AAC AAC ACA AGG GGG GGG GTA TCT AGT TGA CTA GTA CAT GAC CAC TTG A [13]	239
apta#3	No sequence	33.8 ± 7.9

**Table 2:** MD simulation details.

Name	System Composition (Number of Molecules Is Indicated in Parentheses)	Number of Replicas and Productive MD Length, ns	Clustering Cut-Off, nm
apta#1	Aptamer(1)/Water(36541)/Mg^+^(86)/Cl^−^(1)	3 × 150	1.0
apta#2	Aptamer(1)/Water(28807)/Mg^+^(86)/Cl^−^(1)	3 × 150	0.6

**Table 3:** Comparison of apta#1 models generated by productive molecular dynamics with SAXS data.

Model	*χ* ^2^	*R_g_*, nm	*D_max_*, nm	Model	*χ* ^2^	*R_g_*, nm	*D_max_*, nm
1	15.3	3.5	11.8	23	34.4	4.3	14.4
2	25.3	3.3	10.6	24	50.6	4.7	16.1
3	14.0	3.2	10.1	25	15.9	3.3	11.3
4	17.1	3.1	10.3	26	19.1	3.5	11.8
5	40.8	3.8	11.9	27	40.4	4.0	13.2
6	49.5	4.7	17.1	28	19.4	3.5	11.1
7	9.1	3.3	10.8	29	7.2	3.1	10.7
8	61.7	4.1	13.0	30	59.8	4.9	10.6
9	25.6	3.5	11.9	31	35.2	3.5	10.5
10	23.7	3.4	11.3	32	53.6	4.0	12.7
11	4.9	3.1	10.4	33	50.4	3.6	12.7
12	18.1	3.7	12.7	34	27.2	5.0	18.2
13	15.1	3.2	10.0	35	50.4	2.9	9.5
14	54.2	5.2	18.5	36	28.1	3.1	9.6
15	35.0	3.9	12.7	37	23.0	3.3	10.6
16	18.8	3.3	10.5	38	33.8	3.7	11.7
17	15.6	3.1	10.7	39	34.0	3.4	12.0
18	22.2	3.1	9.0	40	23.7	3.7	12.9
19	47.1	5.1	19.4	41	37.8	3.6	10.1
20	34.3	3.6	11.7	42	52.4	3.9	12.3
21	53.8	3.7	11.4	43	43.5	4.2	13.0
22	30.1	5.0	17.8	Ab initio bead model	2.2	3.3	11.5

## Data Availability

Data are contained within the article.
